# Promising Points for Intervention in Re-Imagining Partnered Research in Health Services Comment on "Experience of Health Leadership in Partnering with University-Based Researchers in Canada – A Call to ‘Re-imagine’ Research"

**DOI:** 10.34172/ijhpm.2020.24

**Published:** 2020-02-19

**Authors:** Eugenia Canas, J. Kevin Shoemaker, Anita Kothari

**Affiliations:** ^1^Faculty of Information and Media Studies, Western University, London, ON, Canada.; ^2^School of Kinesiology, Faculty of Health Sciences, Western University, London, ON, Canada.; ^3^School of Health Studies, Faculty of Health Sciences, Western University, London, ON, Canada.

**Keywords:** Health System, Knowledge Translation, Community-University Research, Research-Service Gap

## Abstract

In this commentary, we respond to Bowen and colleagues’ empirical study of research partnerships between Canadian health organizations and university-based investigators. We draw on our experiences of university and health-services partnerships to elaborate on some of the misalignments between researchers and health services leaders identified by Bowen et al. We take up Bowen and colleagues’ call to re-imagine research by proposing three promising points of intervention in research partnerships. These are: (1) orient towards research relationships rather than project-based partnerships; (2) recognize shared and diverging expectations and objectives; and (3) foster a more nuanced understanding of mutual gains.

In their recent article, Bowen et al^1^ invited readers to re-imagine research through a deeper understanding of effective partnerships between health organizations and university-based investigators. They noted that many aspects of effective partnered research remain unexplored, despite growing pressure to engage in partnerships of this nature. Their article — based on empirical findings from interviews with 25 senior health managers in health service organizations — highlighted tensions that undermine the conduct and uptake of research in the design and delivery of health services. Through our experience in similar settings, we characterize these as misalignments between researchers and health service administrators relating to: (*a*) the promise and realities of research; (*b*) the work needs and expectations for these parties; and (*c*) what may be needed in the system now versus what knowledge generation can offer broadly.

Greenhalgh^2^ has eloquently responded to this study by linking to complexity theory as a helpful approach to these divides, thus drawing attention to the context of partnership. We further respond to Bowen and colleagues’ call by specifying areas where re-imagination is overdue, and possible, based on our experiences of partnered research in health services settings. We identify three potential points of intervention: (1) orient towards research relationships rather than project-based partnerships; (2) recognize shared and diverging expectations and objectives; and (3) foster a more nuanced understanding of mutual gains. Although we have identified these three discrete areas, we recognize that they overlap, and that their elements may be serving multiple functions simultaneously.

Our reflection centres on the fundamental assumptions, nuances of collaboration, and structural considerations that have directly impacted our past research partnerships. We write from diverse positions: as an emerging academic with professional experience in community-university engaged research (first author); as a senior basic science researcher with university administration experience (second author), and as an established academic with a long-standing program of research in public health and integrated knowledge translation (third author).

## Our Experiences of Partnered Research

Bowen and colleagues’ findings echo what has often been expressed in the knowledge translation literature, namely that research is experienced as unhelpful or irrelevant to decision-making by many within the system.^3,4^ A key difference in this investigation, however, lies in the depth of the inquiry with senior managers, who operate using both an immediate service-delivery focus and a mid- and long-term vision of what health organizations need in changing political and economic contexts. Their findings resonate with our experiences, where we have often found these two types of partners have different fundamental objectives and expectations relating to research.

In the study, senior managers listed organizational stress and restructuring, and researchers’ limited readiness to work in the fast-paced healthcare environment, as major barriers to partnership. Their expectations of research partnerships and evidence focus on applicability to daily practice *now,* with the aim of supporting staff to do their job well. When research findings do not clearly inform this, or even oppose the status quo, its insights are perceived as unhelpful or irrelevant.

Academic researchers, on the other hand, may have broader and sometimes more diffuse objectives, such as generating knowledge that may serve conceptually for further inquiry, or that eliminates conclusively *what doesn’t work.* In addition to disciplinary norms, the pace of research also responds to an institutional culture, which is often inflexible and sometimes unmanageable. The timelines of funding bodies and ethics review boards, as well as the ebb and flow of (student) human resources, may be out of sync with the pace needed by the healthcare organization.

## Orienting Towards Relationships, not Projects

While these diverse experiences, pace and objectives may look like a divide between university-based investigators and health organizations, we suggest that it is in these points of difference where collaboration finds its unique gains. For this to occur, investigators and healthcare administrators may need to work on sustaining a long-term relationship, rather than on a project-by-project basis.

Long-term research relationships are not without their challenges, and these certainly merit further study. One important hindrance to quality partnerships that we identify is the assumption that research should yield meaningful and actionable results every time. Investigators cannot guarantee that all knowledge will be useful or timely.^5^ What research reveals may in fact be disruptive of the current practice culture of an organization — and it is no less valid or relevant for being so. Research results may often point out exactly what needs to change in practice, but sometimes such change exceeds the scope of what organizational leaders can do. This is common in research that raises the role of structural and social determinants of health in a services context.^6,7^ Such findings often have little application in downstream practice, and require change far beyond what administrators can do. Nonetheless, they are an important message that supports the re-orientation of organizational missions towards particular value systems (namely, equity-oriented).

## Recognizing Shared and Diverging Expectations

In our experience, the greatest support to effective partnerships has been a commitment to recognize, as early as possible, what investigators and organizational leaders expect from research. This clarity reframes differences in culture and pace as features of the partnership landscape that must be navigated, rather than ignored or wished away.We reaffirm Bowen and colleagues’ finding that institutional agreements are needed for effective partnerships: institutional agreements, initiated by the health system administrator and collaboratively revised to reflect the changing organizational context, are a crucial instrument. They set out clear steps for the exchange of data between partners; support the partners in communicating at a consistent and high level even through changes in personnel; give shape to hiring, training and intellectual property approaches; and, introduce accountability mechanisms. The development of institutional agreements might involve difficult conversations, but this is systemic engagement.

Bowen and colleagues’ finding that evaluation and quality improvement are often partitioned from research in organizational settings reveals a missed opportunity that is best harnessed through long-term research relationships, rather than one-off projects. When engaged in the long-term, researchers can deliberately break down these silos by inviting staff from evaluation and quality-improvement departments to become part of the research team. Our past research partnerships, in addition to their intended focus, have often generated a high-level story of how an organization is oriented which has contributed to organizational evaluation efforts. In one of our experiences, a partner organization was under pressure to show outcomes from specific programs in order to secure its sustainability. While the programs were indeed valuable to people experiencing them, the nature of that contribution could not be captured in consistent and significant numbers because the service users are difficult to reach. The research served to describe the human impact of services, and provided a vantage point that complemented how the evaluation team had been approaching their work. This type of research output can empower health services leaders to act and advocate within their larger context.

Another common area of diverging expectations relates how research findings are used. Findings might be communicated to different interested parties through tailored mechanisms, as recommended in the knowledge translation literature: Lay persons might receive an evidence-based narrative, for example, to raise awareness of the issue, while Board members might receive a succinct one pager with recommendations to support decision-making. Peer-reviewed publications may hold zero importance to an organization, while they matter to investigators, who are willing to spend the time necessary on these outputs.

For health organizations, the fast-paced health system environment means that, as Bowen et al point out, the political context might have changed to the extent that the research findings, when available, seem less relevant. When faced with this situation, organizational leaders we have worked with were able to articulate how the research findings might inform the new health system goals. In other words, these knowledge users were able to see the transferability of the findings in a way that we, the researchers, did not immediately pinpoint. As investigators, we have been transfixed on better communication of research but lack a deep understanding of how our research can alleviate political ambiguity — a gap to which our knowledge user partners can be encouraged to contribute.^8^

## Fostering a Nuanced Understanding of Mutual Gains

With Bowen et al, we agree that university investigators need to look to new ways of doing research. This involves re-imagining both how we conduct research, and what we gain by it. On the doing side, to match the rapid pace of health system operations, we need to be more comfortable sharing preliminary or mid-point research findings. These mid-point discussions with organizational leaders could go a long way in influencing final directions, thus increasing the relevancy and impact of research. And, such discussions can help tweak the research in directions that are helpful and relevant. However, this practice will merit reflection before it can become commonplace in robust ways. Sharing preliminary findings requires specific approaches and guidelines for different types of researchers. For example, one challenge with this in the basic quantitative research model is that early information-based on a few data points often is misleading, particularly if the “average” response is the goal. These details may or may not be relevant in a systems research context.

The mutual gains of collaboration may also take forms that have not been envisioned by the university or research funders. Our relationships with health services organizations do take extra time, and this time is not often acknowledged in our promotion, grant competitiveness or tenure conventions. It involves our presence at events that matter to an organization’s identity and growth — such as annual general meetings, public or community events, and social media — and we think this time is well invested. We have found that when we bring our energy to partnerships in this way, they grow naturally and are built on trust, a clearer mutual vision, and the ability to exchange ongoing new knowledge or important political information that support a long-range view for both partners.^5^ In our experience of partnerships, our presence in this way allows health administrators to reach out spontaneously to university-based investigators, whenever other forms of evidence are needed to support practice change. As investigators, being able to call up persons in leadership within a health organization to discuss what really matters in practice at a given time breathes relevance into our research. As noted by Bowen et al, strong executive leadership and multi-system action are needed for strong partnerships ─ and an integral part of that task is for organizations, university and research-funding administrators to see the nuanced value of investing in long-term relationships with knowledge users.

A better understanding of research partnerships includes attention to the multiple levels at which relationships within health services organizations occur. They take place in other contexts too: while significant work currently centres on patient and public involvement, less discussion has focused on industry/corporate partnerships. Research relationships with industry have grown increasingly important in the health services sector. We propose this is a crucial area of future investigation.

In conclusion, we add to Bowen and colleagues’ call to re-imagine research with the specific endorsement of research partnerships based on long-term relationships. We call for a richer understanding of how these long-term relationships unfold, including problematizing and identifying their areas of growth. As we have experienced it, this long-term orientation supports a more nuanced communication about the shared and diverging expectations of research for each party, and of the mutual gains and advantages of collaboration. These advantages include increased use of research evidence and investigators’ support to inform the decision-making of leaders in health organizations, and an overall alignment with the larger call for the democratization of science.

## Ethical issues

Not applicable.

## Competing interests

Authors declare that they have no competing interests.

## Authors’ contributions

All authors were involved in the conception of this commentary. Based on a preliminary team conversation, EC drafted the initial manuscript and subsequently made changes using feedback from JKS and AK. All the authors read and approved the final version of the manuscript.

## Authors’ affiliations

^1^Faculty of Information and Media Studies, Western University, London, ON, Canada. ^2^School of Kinesiology, Faculty of Health Sciences, Western University, London, ON, Canada. ^3^School of Health Studies, Faculty of Health Sciences, Western University, London, ON, Canada.
